# Dimethyl carbonate synthesis from CO_2_ and methanol over CeO_2_: elucidating the surface intermediates and oxygen vacancy-assisted reaction mechanism†

**DOI:** 10.1039/d3sc04466a

**Published:** 2023-11-21

**Authors:** Dragos Stoian, Toshiyuki Sugiyama, Atul Bansode, Francisco Medina, Wouter van Beek, Jun-ya Hasegawa, Akira Nakayama, Atsushi Urakawa

**Affiliations:** a Institute of Chemical Research of Catalonia (ICIQ), The Barcelona Institute of Science and Technology (BIST) Av. Països Catalans 16 43007 Tarragona Spain; b Department of Chemical Engineering, University Rovira i Virgili Av. Països Catalans 26 43007 Tarragona Spain; c Institute for Catalysis, Hokkaido University Sapporo 001-0021 Japan; d Department of Chemical System Engineering, The University of Tokyo Tokyo 113-8656 Japan nakayama@chemsys.t.u-tokyo.ac.jp; e The Swiss-Norwegian Beamlines (SNBL) ESRF – The European Synchrotron Radiation Facility BP 220 F-38043 Grenoble France; f Catalysis Engineering, Department of Chemical Engineering, Delft University of Technology Van der Maasweg 9 2629 HZ Delft The Netherlands A.Urakawa@tudelft.nl

## Abstract

Surface intermediate species and oxygen vacancy-assisted mechanism over CeO_2_ catalyst in the direct dimethyl carbonate (DMC) synthesis from carbon dioxide and methanol are suggested by means of transient spectroscopic methodologies in conjunction with multivariate spectral analysis. How the two reactants, *i.e.* CO_2_ and methanol, interact with the CeO_2_ surface and how they form decisive surface intermediates leading to DMC are unraveled by DFT-based molecular dynamics simulation by precise statistical sampling of various configurations of surface states and intermediates. The atomistic simulations and uncovered stability of different intermediate states perfectly explain the unique DMC formation profile experimentally observed upon transient operations, strongly supporting the proposed oxygen vacancy-assisted reaction mechanism.

## Abstract 2

Oxygen vacancy-assisted reaction mechanisms and key surface intermediates were uncovered by *operando* spectroscopic and DFT-based molecular dynamics simulations in direct dimethyl carbonate (DMC) synthesis from CO_2_ and methanol over CeO_2_ catalyst.

## Graphical abstract

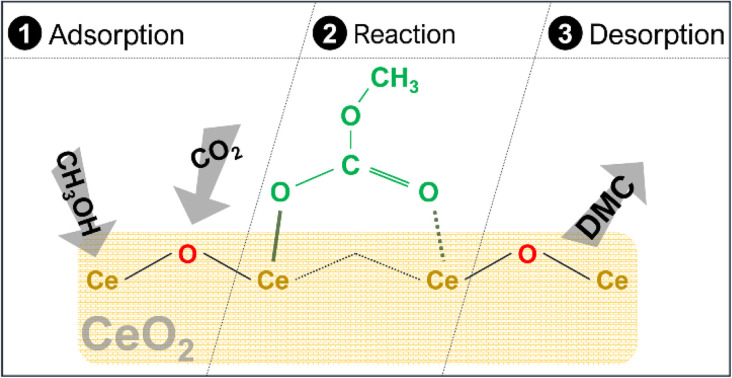

## Introduction

**Scheme 1:**
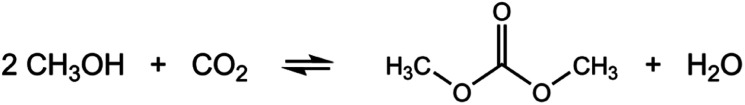
Direct DMC synthesis from CO_2_ and methanol.

The increasing interest in dimethyl carbonate (DMC) has been witnessed in the last decades for its usefulness: polar solvent for paints and coatings, methylating and carbonylating agent in organic synthesis, electrolytes in Li-ion batteries, and an appropriate substitute for methyl *tert*-butyl ether (MTBE) as a fuel additive owing to its high oxygen content.^1–7^ While one of the major synthesis paths of DMC is *via* the reaction of methanol with phosgene (COCl_2_), nowadays the industrially recognized processes involve either oxidative carbonylation reactions using CO (Enichem, Ube and Bayer AG), trans-esterification reactions of organic carbonates with methanol (Asahi Kasei), or a two-step urea methanolysis method developed by Catalytic Distillation Technologies, Inc.^8^ Despite these improvements, it can be easily understood why a safer, more eco-efficient and economic pathway for DMC synthesis is desired. The development of the direct reaction between CO_2_ and short chain alcohols, particularly methanol (MeOH) and ethanol (EtOH), for the synthesis of organic carbonates such as DMC or diethyl carbonate (DEC) (Scheme 1, DMC synthesis) has drawn great attention in the light of urged CO_2_ chemical fixation, green chemistry (high atom efficiency processes), and safety by replacing the highly toxic and corrosive phosgene molecule by CO_2_ as carbonyl source.

In the last 20 years, various homogeneous and heterogeneous catalysts were reported for the direct carboxylation reaction of methanol using CO_2_. Heterogeneous catalysts are more widely investigated and different materials have been tested under both batch and continuous operation. Among them, zirconia (ZrO_2_), ceria (CeO_2_), and ZrO_2_–CeO_2_ solid solutions^9–11^ have been reported as the most effective catalysts in the direct DMC synthesis, while other catalyst materials afforded no or very little formation of DMC.^12^ Generally, the presence of both acidic and basic sites is required for the activation of CO_2_ and methanol. Indeed, the aforementioned reported active catalysts possess both the acidic and basic properties.^13^ Nevertheless, in practice the reaction is highly thermodynamically limited and the DMC yield can be enhanced only up to about 1% even under thermodynamically favourable high-pressure conditions (*ca.* 400 bar).^14^ Recently, a drastic improvement in DMC yield (>90%) was reported by Tomishige *et al.* over CeO_2_ as the unique catalyst through *in situ* dehydration where 2-cyanopyridine (2-CP) serves as organic dehydrating agent to remove the water byproduct (Scheme 1). CeO_2_ functions as catalyst in both DMC synthesis and dehydration reaction optimally at *ca.* 120 °C. This strategy has been demonstrated for batch as well as continuous operations.^14–17^ It is noteworthy that the rate determining step of the DMC synthesis is unaltered by the presence of 2-CP. This implies that understanding the simpler reaction system without the dehydrating agent would be of direct relevance in understanding more complex yet more practical system with the dehydrating agent.^16^

The unique catalytic performance of CeO_2_ in this reaction may originate from the synergy between its acid-base and redox properties. Izumi *et al.* studied the reducibility of CeO_2_ promoted with Cu (0.1 to 0.5 wt%) by X-ray absorption spectroscopy (XAS) after high temperature reduction treatment (400 °C) and subsequent CO_2_ adsorption. They suggested that the partial reduction of Ce^4+^ sites to Ce^3+^ may be beneficial for DMC formation.^18^ Also, Aresta *et al.* correlated by *ex situ* X-ray photoelectron spectroscopy (XPS) the origin of catalyst deactivation, thus the activity, with the oxidation state of Ce.^19,20^ In a more recent study, Li *et al.* synthesized Zr-doped CeO_2_ nanorods, and they linked the DMC production rate to the highest number of oxygen vacancies (*i.e.* extracted from *ex situ* Raman and XPS measurements; *versus* bare CeO_2_) that allows an increased CO_2_ adsorption rate.^21^ Despite all these indications, the contribution and importance of the redox properties or surface defects of CeO_2_ in the reaction has not been proven under working (*operando*) reaction conditions. Particularly, the reaction is commonly performed at relatively low temperatures (100–150 °C) where the reduction of surface and bulk CeO_2_ is unlikely to occur according to temperature programmed reduction studies^22–24^ and the detection of electronic structure change of Ce, if any, is expected to be challenging. Furthermore, no *operando* spectroscopic investigation has been reported bridging the type of surface species present under reaction conditions with DMC formation by simultaneous detection of the product concentration.

Following this background, in this work we elucidate the surface chemical intermediate, at its energetically resting state, leading to DMC formation by means of *operando* diffuse reflectance infrared Fourier transform spectroscopy (DRIFTS) coupled with multivariate spectral analysis^25–27^ which enables unreferenced spectral separations (ESI, Fig. S1†). Furthermore, through density functional theory (DFT) calculations, XAS and Raman spectroscopy, the critical roles of surface oxygen vacancies of CeO_2_ at the rate limiting step are uniquely unravelled.

## Results and discussions

**Fig. 1:**
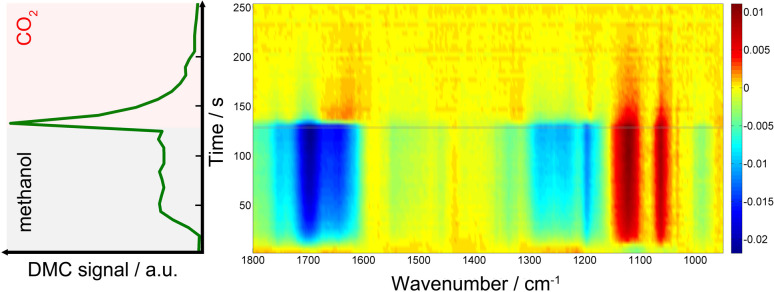
(left) MS signal of DMC (*m*/*z* = 59) and (right) time-resolved DRIFT spectra taken during methanol (the first half period) *vs.* CO_2_ (the second half period) concentration perturbation experiment performed at 120 °C at 7 ml min^−1^. The time-axes (*y*-axis) of both figures are identical. The DRIFT spectra were calculated taking the last spectrum in the CO_2_ atmosphere as background.

Fig. 1 presents time-resolved *operando* DRIFT spectra and DMC concentration profile under a periodic concentration change of methanol (16.5 vol% in He, the first half period of 128 s) and CO_2_ (the second half period of 128 s) performed at 120 °C (averaged over 8 periods to improve S/N). The MS signal (Fig. 1, left) shows that DMC was formed under the studied condition. The production level of DMC increased upon switching from CO_2_ to methanol, reaching a constant level after *ca.* 40 s. Interestingly, the DMC production was boosted by *ca.* 4 times upon switching from methanol to CO_2_ and then gradually ceased with time, almost completely under the flow of CO_2_. The stable activity after 40 s is at the same level as that of the steady-state activity of DMC formation by passing MeOH + CO_2_ (ESI, Fig. S3†). This means that MeOH *vs.* CO_2_ perturbation creates favourable condition for DMC formation, enhancing the formation rate by 4 times compared to the steady-state activity. The differential DRIFT spectra in the low frequency region from 950 to 1800 cm^−1^ (Fig. 1, right; the last spectrum of the period, *i.e.*, at the end of the CO_2_ period, was used as the background) shows the characteristic C–O stretching vibrations of bridged and terminal methoxy species appearing at *ca.* 1060 and 1120 cm^−1^, respectively,^10^ as positive bands (in red) when the atmosphere was switched to methanol. Likewise, the absorbance of the methoxy bands (hence the concentration of methoxy species) for the MeOH + CO_2_*vs.* CO_2_ experiment is *ca.* 4 time less compared to the case of MeOH *vs.* CO_2_, well in-line with the semi-quantitative analysis of the MS profiles and the observation from Fig. 1, left which tells us that methanol adsorption needs to take place first in order to activate the DMC formation (ESI, Fig. S4† for more detailed discussion).

At the same time, several negative bands characteristic of carbonates/bicarbonates were observed in the region from 1150–1800 cm^−1^ under the flow of MeOH. According to literature, these bands originate from carboxylate and protonated carboxylate species on the surface of the catalyst (1695 cm^−1^), bridged (bi)carbonates (1235 and 1645 cm^−1^), and monodentate and bidentate carbonates (1336, 1458 cm^−1^ and 1282, 1548 cm^−1^, respectively).^10,28–31^ The results clearly indicate that carbonates/bicarbonates formed under CO_2_ atmosphere were replaced (thus the bands appear negative) by the methoxy species under methanol atmosphere, and *vice versa* under CO_2_ atmosphere. At first glance, the DRIFT spectra do not show signatures of transient surface species whose concentration is similar to that of DMC, except a few slightly positive signals at *ca.* 1310 and 1630 cm^−1^ appearing upon switching to CO_2_. This sort of ambiguousness in spectral analysis is often encountered and arises from overlapping peaks and also impossibility to deconvolute the spectral contributions of different chemical species due to the unavailability of proper reference spectra of the chemical species that are only present/detected under reaction conditions and/or under transient conditions.

**Fig. 2:**
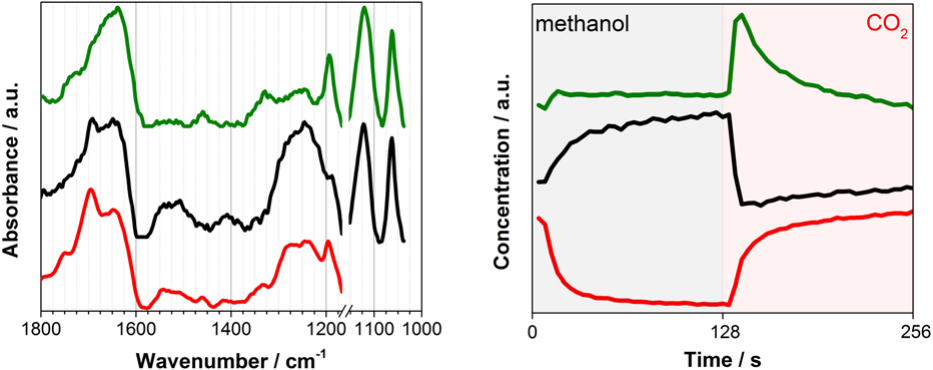
(left) Three components spectra and (right) the corresponding concentration profiles obtained by multivariate spectral analysis applied on the DRIFT spectra shown in Fig. 1 (the first half is methanol and the second half is CO_2_ atmosphere). To ease the data analysis and improve component separation, the spectral analysis has been performed on two separate regions of methoxy (below 1170 cm^−1^) and carbonates (above 1180 cm^−1^). Both spectra and concentration profiles are normalized for clarity.

To overcome this limitation, multivariate spectral analysis was employed to disentangle overlapping peaks into the spectra of “kinetically pure” components (*i.e.*, surface species). Besides the spectral separation, it yields conveniently the corresponding concentration profiles of the components. Provided that the data quality is good in terms of S/N, the multivariate spectral analysis can overpower another famous technique used for detection of minor species, *i.e.*, modulation excitation spectroscopy (MES, using phase sensitive detection), especially when there is a high degree of spectral overlap and species with extremely different kinetics are involved. For more details and comparison between the multivariate spectral analysis and MES, the reader is redirected elsewhere.^26,32–34^Fig. 2 shows the results of the multivariate spectral analysis on the DRIFT spectra shown in Fig. 1. For comparison, the results obtained *via* MES analysis are included in the ESI, Fig. S5.† The analysis identified three kinetically distinct components. Most strikingly, the analysis could separate the spectral component (Fig. 2, green) with the concentration profile perfectly matching with that of DMC (Fig. 1, left). The other two components show characteristic features of (i) methoxy species (Fig. 2, black) and (ii) carbonate/bicarbonate species (Fig. 2, red). The corresponding concentration profiles of these components (Fig. 2, right) obviously reflect the atmosphere of the gas phase.

*In situ* infrared and Raman studies reported by Tomishige, Bell and their co-workers over ZrO_2_ materials suggested the formation of monomethyl carbonate (MMC, CH_3_O–COO–Zr) as possible intermediate species through the reaction between methoxy species (CH_3_O^−^) and CO_2_.^35–37^MMC further reacts with methanol (methoxy) to afford DMC. Later, it was indicated that the reaction proceeds *via* the same intermediate (*i.e.*MMC) over CeO_2_ catalysts.^10,16^ There are also reports supporting the existence of a different intermediate like carbomethoxide (CH_3_OCO–Ce) species as suggested by Wang *et al.*^28^ The spectral characteristics of the captured intermediate (Fig. 2, green) whose concentration profile resembles that of gas phase DMC generally show the mixed features of methoxy (1060 and 1120 cm^−1^) and (bi)carbonates (*e.g.* 1200–1800 cm^−1^), although they are somehow different especially in the region of 1200–1600 cm^−1^ where the characteristic bands of MMC are expected to appear according to the literature.^10,20,28,38^ More precisely, Lavalley *et al.* assigned the two bands we observed at *ca.* 1330 and 1460 cm^−1^ to a coupling between the bending mode of CH_3_ and the stretching vibration of OCO.^39^ This clearly indicates that the captured intermediate is MMC or species alike. The spectral features of the intermediate (Fig. 2, green) in the methoxy region appear similarly to those of methoxy species (Fig. 2, black). A closer look verifies that they are similar but there are noticeable differences especially in the band of terminal methoxy at *ca.* 1120 cm^−1^ of the intermediate, showing a broadening and a small shift towards lower vibrational frequency (ESI, Fig. S2†). This implies that the terminal methoxy has reacted or is interacting with the CO_2_ molecule trapped on the surface, resulting in the red shift. Also, intriguingly the species responding to the gas-phase methanol concentration (Fig. 2, black) show the spectral features of (bi)carbonates in the region 1200–1800 cm^−1^. This is indicative of the formational correlation of methoxy species with specific surface (bi)carbonate species. The dissociation of MeOH into MeO and H is known^10^ to take place upon methanol adsorption over CeO_2_ and this surface adsorbed H may lead to more pronounced formation of bicarbonates from carbonates (see the discussion below for DFT calculations) and the spectral feature of the methoxy species may reflect the accompanied formation of bicarbonate surface species since the multivariate spectral analysis cannot disentangle spectral components behaving kinetically identical. A similar discussion for the high frequency region (above *ca.* 2000 cm^−1^) is presented in the ESI (Fig. S6 and S7†) including the difference DRIFT spectra and the corresponding multivariate spectral analysis results.

**Fig. 3:**
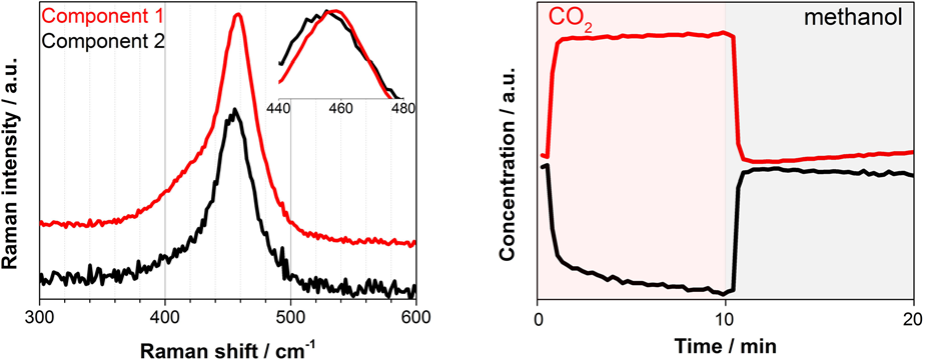
Multivariate spectral analysis applied for F_2g_ band of CeO_2_ during DMC synthesis experiment (CO_2_*vs.* methanol) at 140 °C. The Raman measurement was performed with a 532 nm laser (green). Both spectra (left) and concentration (right) profiles are normalized for clarity.

Under similar transient conditions we looked into the change in the oxidation state of Ce by XAS at Ce K and L3-edges and structural changes by Raman spectroscopy. Under oxidation (O_2_) and reduction (H_2_) treatment at high temperature (350 °C), the multivariate spectral analysis on the XAS and Raman data could clearly extract the redox features of CeO_2_ (ESI, Fig. S8–S10†), whereas these redox features could not be observed at the reaction temperature of 100–150 °C which was obviously too low for the redox to take place as expected. Although we could not detect spectral changes in the bulk-sensitive XAS under the transient reaction conditions of MeOH *vs.* CO_2_ flow, there was a clear change in the F_2g_ Raman active mode at *ca.* 460 cm^−1^, corresponding to the symmetric breathing of O^2−^ atoms vibrations around the Ce^4+^ cations (Fig. 3). The width and position of this band is known to be extremely sensitive to any structural disorder of the O-sublattice.^40^ Multivariate spectral analysis on the Raman data disentangled the two spectral components with contrasting concentration profiles: one increasing upon CO_2_ exposure which is obviously assigned to the band associated with the Ce^4+^ state (Fig. 3, Component 1, red), and the other increasing with methanol with the band feature slightly red-shifted and broadened (Fig. 3, Component 2, black). These changes are in accordance with the reports on CeO_2_-based materials under reduction–oxidation cycles.^41–43^ Yoshimura *et al.* use the changes in Raman-allowed F_2g_ mode (*i.e.* shifts to lower energies, and the line shape getting progressively asymmetric with the crystal size decrease) in a study about the identification of defects in ceria-based nanocrystals by UV resonance Raman spectroscopy.^44^ This subtle change took place on the surface of the material and therefore could not be measured by XAS. Likewise, the change was less obvious when a longer wavelength excitation laser (785 nm) was used in Raman since in this case we sample more bulk of the material (ESI, Fig. S11†). Given the magnitude of the spectral change and the mild reaction conditions it becomes rather difficult to state the existence of a complete Ce^4+^ ⇔ Ce^3+^ cycle despite a recent publication by Tomishige *et al.* which reported the redox properties of CeO_2_ catalysts in organic reactions (*i.e.* synthesis of imines from alcohols and amines) at temperature as low as 30 °C.^45^ In literature, Mullins *et al.* probed the surface sites of CeO_2_ nanocrystals with well-define surface planes *via* methanol adsorption, and they observed slight reduction of the rods (110) and cubes (100) surfaces at room temperature by a UV Raman study. In addition, surface science reports on methanol adsorption over thin-film model CeO_2_ catalyst support the reduction phenomenon occurring in the topmost oxide layers even at ambient or sub-ambient temperatures (Mullins *et al.* and Skala *et al.*).^46–48^ Based on what has been reported, the creation of a nonstoichiometric CeO_2−*δ*_ surface *via* the formation of some surface and subsurface O-vacancies (*i.e.* defective surface) is possible and explains the Raman results. Such defective surface formation would lead to a more electron deficient or more electron rich CeO_2_ state depending on the gas atmosphere. Glatzel *et al.* have previously invoked this picture of an electron sponge for CeO_2_ nanoparticles studied by HERFD-XAS.^49^ They found an increase of the interatomic distances between Ce and O during the catalytic decomposition of hydrogen peroxide while stating that the redox partner is not a local Ce^3+^ site, but the electron density that is received and released during the reaction is delocalized over the atoms of the nanoparticles. Furthermore, the emergence of two bands in the 2000–2250 cm^−1^ region of the DRIFT spectra that can be linked to Ce^3+^ surface sites formation induced by methanol adsorption over CeO_2_ can further support the Raman results and thus methanol adsorption-induced Ce^3+^ formation (ESI, Fig. S6†) which is important for the enhanced DMC formation (Fig. 1, left, Fig. S3†).

The mechanistic investigation by DFT calculations strongly supports our experimental observations and clarifies atomistic and electronic insights. The computational details are described in ESI.† While methanol adsorption occurs preferentially *via* the dissociation of the molecule into CH_3_O^−^ and H^+^, the favoured and most stable adsorption state of CO_2_ is represented by a monodentate carbonate species involving a covalent bond between the C atom of the CO_2_ molecule and surface O atoms belonging to CeO_2_ (see ESI, Fig. S12† for adsorption structures).

**Fig. 4:**
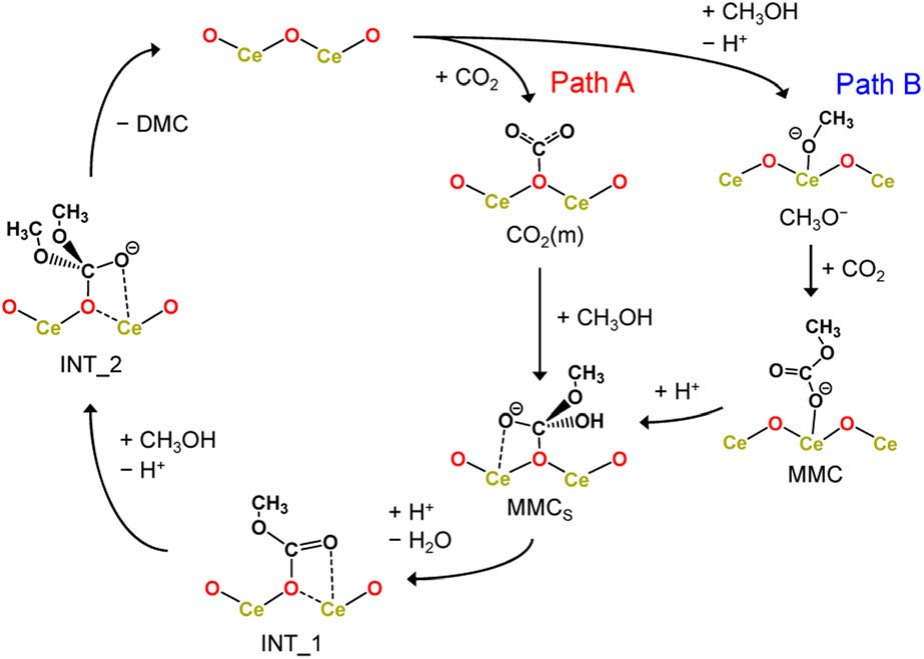
Reaction mechanism of DMC formation from CO_2_ and methanol. Path A starts with an adsorbed CO_2_ molecule while Path B starts from the dissociative adsorption of a methanol molecule.

Here, we considered the two reaction pathways for DMC formation, one starting from an adsorbed CO_2_ molecule that is a monodentate carbonate species, CO_2_(m) (Path A), and the other starting from the dissociative adsorption of a methanol molecule (Path B), as shown in Fig. 4. In Path A, this monodentate structure is attacked by the methoxy species (CH_3_O^−^), leading to the formation of surface monomethyl carbonate species (MMC_S_). After removal of the hydroxyl group by accepting a proton, an intermediate species (INT_1) is formed by releasing a water molecule. Subsequently, another methoxy moiety attacks the carbon atom, forming an intermediate structure INT_2, and then the carbon and surface oxygen bond is broken to release a DMC molecule. In Path B, a CO_2_ molecule is inserted into the adsorbed methoxy species, leading to the formation of monomethyl carbonate (MMC) species. This MMC species is nucleophilically attacked by the surface oxygen atom, which results in the formation of MMC_S_ by accepting a proton from nearby methanol molecule. The subsequent reaction pathway from MMC_S_ is the same as that in Path A.

**Fig. 5:**
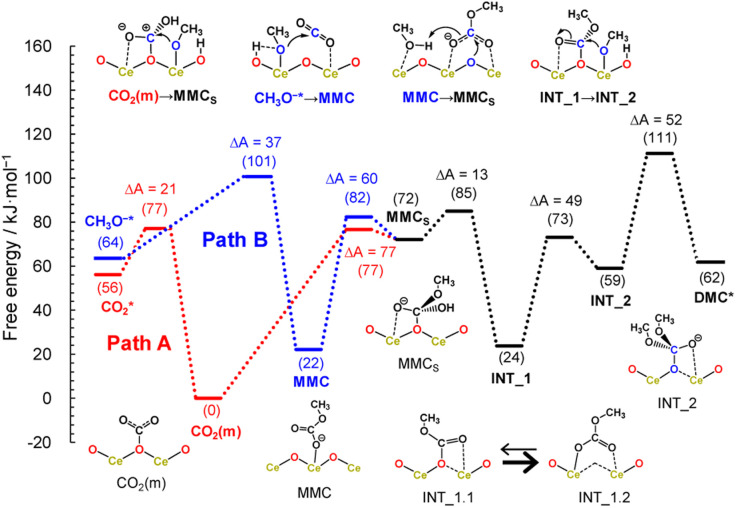
Free energy profiles along the two reaction pathways. The free energy of the monodentate structure CO_2_(m) is set to zero, and relative energy is shown in parenthesis. Activation energy (Δ*A* in kJ mol^−1^) in each step is also included. The inset on the bottom (INT_1 = INT_1.1 → INT_1.2) evidences the key step in the stabilization of the reaction intermediate and formation of an O-vacancy in the catalytic cycle over CeO_2_.

Fig. 5 shows the free energy profiles along the reaction pathways. In the first step of Path A, the CO_2_ molecule is adsorbed on the CeO_2_ surface with a small barrier of 21 kJ mol^−1^ (CO_2_* → CO_2_(m)), forming a monodentate structure CO_2_(m). Then, the surface monomethyl carbonate species (MMC_S_) is generated with an activation energy of 77 kJ mol^−1^ (CO_2_(m) → MMC_S_). Before reaching to this transition state configurations, we find that a proton is transferred from a neighbouring methanol molecule to the surface carbonate species, resulting in the bicarbonate species. This bicarbonate species is attacked by methoxy moiety to form MMC_S_ structure (see the inset of CO_2_(m) → MMC_S_ in Fig. 5 and the snapshot near the transition state structure is shown in ESI, Fig. S13†). The resulting MMC_S_ structure is less stable, and the carbomethoxy species (INT_1.1) is formed with a small barrier of 13 kJ mol^−1^ by eliminating a water molecule (snapshot near the transition state structure is also provided in ESI, Fig. S13†). This carbomethoxy configuration is meta-stable and it readily changes its configuration with a more stable monomethyl carbonate state accompanied by the creation of an oxygen vacancy during the MD simulation (shown as INT_1.2, and snapshot is provided in ESI, Fig. S13†). This intermediate is further attacked by another methoxy moiety, leading to the formation of INT_2 with an activation barrier of 49 kJ mol^−1^. During this attack of methoxy species, the surface vacancy is refilled by the oxygen atom. The final step is a bond cleavage between the carbon and surface oxygen atom, and this step requires an activation energy of 52 kJ mol^−1^. The free energy difference between INT_1 and the transition state between INT_2 and DMC* is estimated to be 87 kJ mol^−1^, which is comparable to the experimental value of 73 kJ mol^−1^ reported in ref. 16.

Next, the reaction mechanism of Path B is analysed in detail. In the first step, the monomethyl carbonate species is formed by the reaction between a dissociated methanol and CO_2_ with an activation barrier of 37 kJ mol^−1^ (CH_3_O^−^* → MMC). The conformation of MMC species during the MD simulation is close to the adsorption structure of DM1 or DM2 shown in ESI, Fig. S12,† where in the MD simulations the hydrogen bond of the carbonyl group is observed with hydrogen in the methanol molecule (snapshot is provided in ESI, Fig. S13†). The next step is a nucleophilic attack of the surface oxygen atom to the carbon atom accompanying a proton transfer to the carboxylate group (MMC → MMC_S_). This process requires activation energy of 82 kJ mol^−1^.

The gaseous product (DMC) profile (Fig. 1) is in good agreement with the mechanistic insights obtained by the DFT calculations. Upon switching from CO_2_ to methanol, there is an induction period before reaching a stable DMC production. This clearly indicates a strong CO_2_ adsorption to the surface sites; it takes some time to replace the strongly adsorbed CO_2_ molecules by methanol. This competitive adsorption and replacement by methoxy retards the DMC formation and also implies that the methoxy and Ce^3+^ generation are crucial for the reactivity towards DMC formation. Also, the spectral features of bicarbonates in the region 1200–1800 cm^−1^ in the methanol atmosphere (Fig. 2, black) correspond to the bicarbonate species before the formation of MMC_S_ structure. On the other hand, upon switching from methanol to CO_2_ there is an immediate formation of DMC with a boost in productivity, demonstrating that for the CO_2_ molecules it is much easier to kick out the previously adsorbed methanol from the surface through MMC intermediate. After that, DMC production ceases quite fast suggesting a very strong CO_2_ absorption to CeO_2_ surface and the lack of any methoxy species in the proximity. Based on the discussion above, we can also conclude that the stability of the intermediate (INT_1.2) and the creation of a defective surface is the key to make CeO_2_ an efficient catalyst for the direct synthesis of DMC while the reaction invokes the image of a Langmuir–Hinshelwood type of mechanism with methanol and CO_2_ molecules adsorbed in the immediate vicinity of a Ce–O pair.

## Conclusions

In summary, the present study describes the *in situ* creation of a defective CeO_2_ surface and its involvement in the gas phase DMC synthesis from CO_2_ and methanol by using a combination of experimental and theoretical tools. While methanol seems to increase the electron density around Ce sites, CO_2_ behaves as an oxidizing agent leading to fully oxidized Ce^4+^. Furthermore, a boost in DMC production is observed when CO_2_ is passed over methoxy covered surface. The surface intermediate species directly correlated with DMC formation was elucidated by *operando* DRIFTS with the aid of the multivariate spectral analysis. It shows the feature of both methoxy and (bi)carbonates, in good agreement with the reported structure of monodentate methyl carbonate. We demonstrate, for the first time ever, the reconfiguration of this species on the CeO_2_ surface *via* a vacancy-assisted mechanism, observation which correlates well with the *operando* Raman results.

Ultimately, direct DMC synthesis from methanol and CO_2_ is un-equivocally driven forward by the presence of neighbouring acid–base pairs over CeO_2_ surface. Nevertheless, the influence of O-vacancies in the reaction mechanism and the extraordinary redox (surface) properties of this oxide cannot be neglected anymore. It has already been shown that an increased surface reducibility which can ease the formation of surface defect sites – responsible for the intermediate stabilization – plays critical roles in the overall reaction mechanism involving the *in situ* water removal by 2-CP molecules (while the DMC formation mechanism is unchanged by the dopants and mainly catalysed by CeO_2_ surface).^50^ Hence, this study establishes new directions for designing better and more stable catalysts for this newly implemented and highly active route to DMC synthesis by direct methanol carboxylation reaction.

## Data availability

Data will be made available on request.

## Author contributions

D. S. performed all experiments and T. S. performed the DFT-based calculations. A. B. and W. v. B. supported synchrotron X-ray-based experiments. F. M., J. H., A. N. and A. U. conceived and supervised this work. D. S., T. S., A. N. and A. U. analysed the data. The manuscript was drafted with the support and contribution from all authors.

## Conflicts of interest

There are no conflicts to declare.

## Supplementary Material

SC-014-D3SC04466A-s001
