# Supplementary material for: Dimethyl carbonate synthesis from CO2 and methanol over CeO2: elucidating the surface intermediates and oxygen vacancy-assisted reaction mechanism
Source: Chem Sci. 2023 Nov 21;14(47):13908–14. doi: 10.1039/d3sc04466a (PMC10699564; doi:10.1039/d3sc04466a)
Supplement: SC-014-D3SC04466A-s001 [file SC-014-D3SC04466A-s001.pdf]

Electronic Supplementary Material (ESI) for Chemical Science.
This journal is © The Royal Society of Chemistry 2023

Supporting Information

Dimethyl carbonate synthesis from CO_2 and methanol over CeO_2: Elucidating the surface intermediates and oxygen vacancy-assisted reaction mechanism

Dragos Stoian[a], [b], [e], Toshiyuki Sugiyama[c], Atul Bansode[a], [f], Francisco Medina[b], Wouter van Beek[e], Jun-ya Hasegawa[c], Akira Nakayama*, [c], [d] and Atsushi Urakawa*, [a], [f]

[a] Dr. D. Stoian, Dr. A. Bansode, Prof. A. Urakawa

Institute of Chemical Research of Catalonia (ICIQ), The Barcelona Institute of Science and Technology (BIST), Av. Països Catalans 16, 43007 Tarragona, Spain

[b] Dr. D. Stoian, Prof. F. Medina

Department of Chemical Engineering, University Rovira i Virgili, Av. Països Catalans 26, 43007 Tarragona, Spain

[c] T. Sugiyama, Prof. J. Hasegawa, Prof. A. Nakayama

Institute for Catalysis, Hokkaido University, Sapporo 001-0021, Japan

[d] Prof. A. Nakayama

Department of Chemical System Engineering, The University of Tokyo, Tokyo 113-8656, Japan, **E-mail: nakayama@chemsys.t.u-tokyo.ac.jp**

[e] Dr. D. Stoian, Dr. W. van Beek

The Swiss-Norwegian Beamlines (SNBL), ESRF - The European Synchrotron Radiation Facility, BP 220, F-38043 Grenoble, France

[f] Dr. A. Bansode, Prof. A. Urakawa

Catalysis Engineering, Department of Chemical Engineering, Delft University of Technology, Van der Maasweg 9, 2629 HZ Delft, The Netherlands, **E-mail: A.Urakawa@tudelft.nl**

Reagents and experimental setup

High surface area CeO_2 powder (>100 m^2/g) was kindly supplied by Daiichi Kigenso Kagaku Kogyo Co. Ltd., Japan and used without any further treatment. Methanol (>99%, HPLC grade) was purchased from Sigma Aldrich. High purity He (>99.999%) and CO_2 (>99.9993%) purchased from Abelló Linde gas was used. A setup similar to that already presented in the literature was employed for the *in situ/operando* XAS/Raman/MS measurements.[1] Experiments were performed at the Swiss Norwegian Beam Line (SNBL) at the European Synchrotron Radiation Facility (ESRF) in Grenoble, France. More information about the optics, beamline layout, monochromators design, and Raman probe design can be found in literature.[2,3] Gaseous CO_2 and He were passed to the cell by means of mass flow controllers. The He flow was saturated with methanol and thus about 16.5 vol% of methanol was contained in the flow.

Operando DRIFTS-MS setup: all experiments were performed on a Bruker Vertex 70V IR spectrometer equipped with a liquid-nitrogen-cooled mercury cadmium telluride (LN-MCT) detector with a home-made temperature controlled stainless steel cell mounted in a Praying Mantis optical accessory (Harrick). The DRIFTS cell mimics the action of plug flow and the design is described elsewhere.[4] The measurement was performed at the center of the catalyst bed consisting of CeO_2. The Praying Mantis accessory consists of an optical system equipped with a series of mirrors for redirection and collection of diffuse reflected light as well as minimizing the detection of specularly reflected light. Spectra were collected at 4 cm^{-1} resolution.

Operando Raman-MS: experiments were carried out using Renishaw inVia spectrometer with 532 nm excitation laser (at SNBL, ESRF) as well as using a BWTEK dispersive i-

Raman portable spectrometers equipped with 532 and or 785 nm excitation lasers and a TE-cooled linear array detector. A gas manifold was used to feed the reactants to the reaction cell (quartz capillary reactor (Hilgenberg GmbH) with 0.7 mm OD with 10 µm wall thickness) and the heating system consisted of a commercially available high temperature (up to 500 °C) hot air gun.

Multivariate curve resolution

Multivariate curve resolution (MCR) has been used in the last 40-50 years and has gained increasing attention due to its excellent ability to deliver the pure component spectra with corresponding response profiles (e.g. pH profiles, time profiles, elution profiles) of the chemical constituents or species of an unresolved mixture when no previous information is available about the nature and composition of these mixtures.[5] Thus, MCR is often used as a "blind-source" spectral separation method (without having any reference spectra *a priori*) as well as an efficient mean to process large data sets coming from the *in situ* measurements performed in labs and at synchrotron facilities all over the world.[1] Herein, a brief description of the MCR-ALS (Multivariate Curve Resolution Alternating Least Square) method is presented. A more detailed description of the method and software used can be found in various literature reports and tutorials. [6–14]

For MCR (**Figure S1**) analysis of a multicomponent system, two main requirements must be fulfilled, namely (i) the experimental data should be structured as a two-way data matrix (or a multiset structure) and (ii) this data set should be explained well enough by a bilinear model using a limited number of components. The MCR bilinear model is usually described by the equation $\mathbf{D} = \mathbf{C}\mathbf{S}^T + \mathbf{E}$, where **D** is the raw data set (initial spectroscopic data table; the rows of matrix **D** are the spectra measured during the experiment), and $\mathbf{S}^T$

(rows) and **C** (columns) are the matrices corresponding to the pure spectra and the related concentration profiles, respectively, for each of the compounds encountered in the system (the superscript T denotes the transpose of matrix **S**). **E** is the matrix of residuals and ideally it should be close to the experimental error.

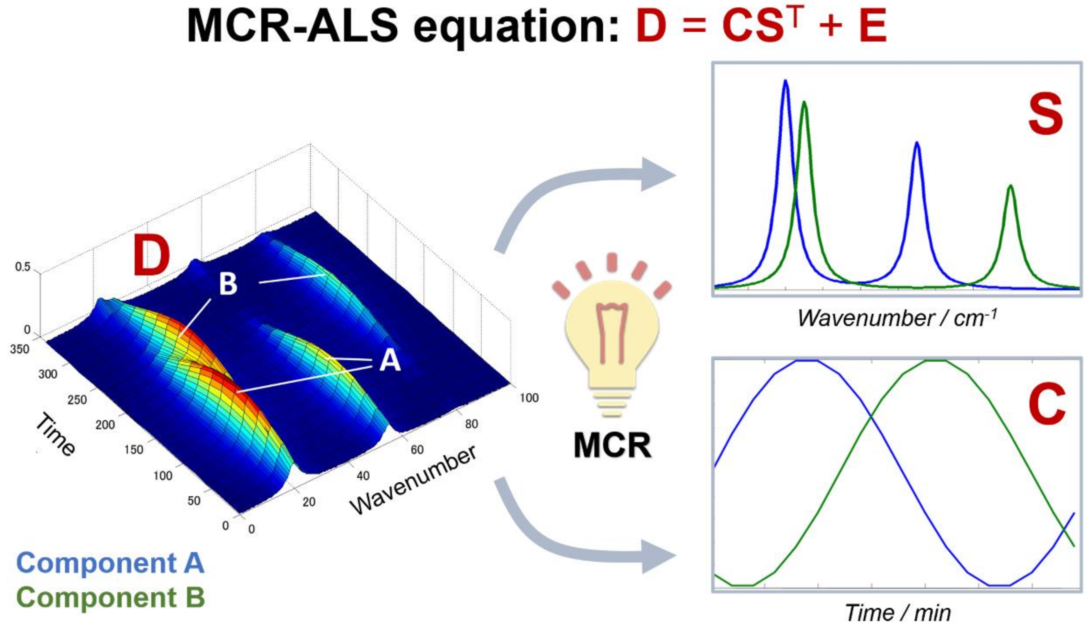

Figure S1 Multivariate curve resolution (MCR) – efficiently solving the mixture analysis problem (e.g. spectroscopic data set).

With the purpose of improving the data treatment and solve the MCR bilinear model and to realistically interpret and extract the profiles of **C** and $\mathbf{S}^T$, a constrained Alternating Least Squares (ALS) algorithm is also implemented. MCR-ALS solves iteratively the equation presented in **Figure S1** by using the Alternating Least Squares algorithm which calculates the concentration and pure spectra matrices to optimally fit the experimental data matrix. To carry out the optimization procedure, a series of steps must be followed, namely (i) estimation of the initial number of components in the system (can be done

either manually or by using single value decomposition algorithm), (ii) preliminary estimation of both **C** and $\mathbf{S}^T$ (realized manually or by means of using the evolving factor analysis method or by a purest variable detection method), (iii) the choice of constraints: several constraints can be applied to model the shapes of the **C** and $\mathbf{S}^T$ profiles (separately), such as non-negativity, unimodality, closure, trilinearity, selectivity or/and other shape or hard-modeling constraints, and (iv) ALS optimization: convergence is achieved after a predefined number of iteration cycles or when there is no longer a visible difference in the standard deviations of the residuals between two consecutive iterative cycles.

In the end, the MCR-ALS algorithm is a very flexible mechanism which, by proper selection and application of the constraints that are fully fulfilled by the raw data set, can deal with huge amounts of datasets resulted from the most diverse situations. The final goal is to learn more about a certain chemical system behavior and to come up with meaningful results from both mathematical and chemical point of view.

IR results from MCR-ALS on the methoxy region

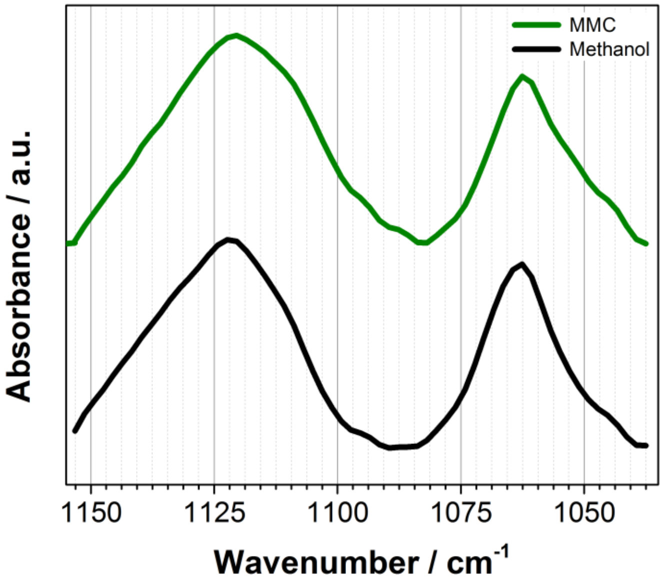

Figure S2 Methoxy region (shown for clarity) of the two components (black, resulting from methanol and green, the component leading to DMC formation) obtained by MCR analysis applied on the DRIFT spectra shown in **Figure 1**.

Semi-quantitative interpretation on DMC amount formation: MS and DRIFTS results

The **Figure S3** shows a semi-quantitative comparison of the amount of DMC formation in three cases, namely (i) MeOH vs. CO_2, (ii) MeOH+CO_2 vs. CO_2 and (iii) MeOH+CO_2 vs. MeOH concentration perturbation experiments. It is important to note the amount of DMC formation, when both reactants are present (the DMC signal at ca. 100 s), is similar for cases (ii) and (iii) as expected. Compared to this steady-state amount of DMC formation, the transient reactivity towards DMC formation upon switching from MeOH to CO_2 during case (i) is nearly 4 times higher. This shows that the surface state, i.e. coverage of surface species and state of Ce, affects the DMC formation drastically. Another interesting observation is that the steady DMC formation amount during the MeOH phase in case (i) is at a similar level to that of (ii) and (iii) during the period when both MeOH and CO_2 are present. This means that CO_2 adsorbed during the CO_2 phase in (i) cannot be reacted quickly and methanol adsorption needs to take place first to activate the DMC formation. As shown previously concerning **Figures 1** and (later on) **S6** (for the high frequency region, see below), the Ce^{3+} and methoxy species grow rather slowly, likely due to the competitive adsorption CO_2 as carbonates which block the adsorption sites of the CeO_2 surface. This competitive adsorption and replacement by methoxy delays the DMC formation and also implies that the methoxy and Ce^{3+} formations are crucial for the reactivity towards DMC formation.

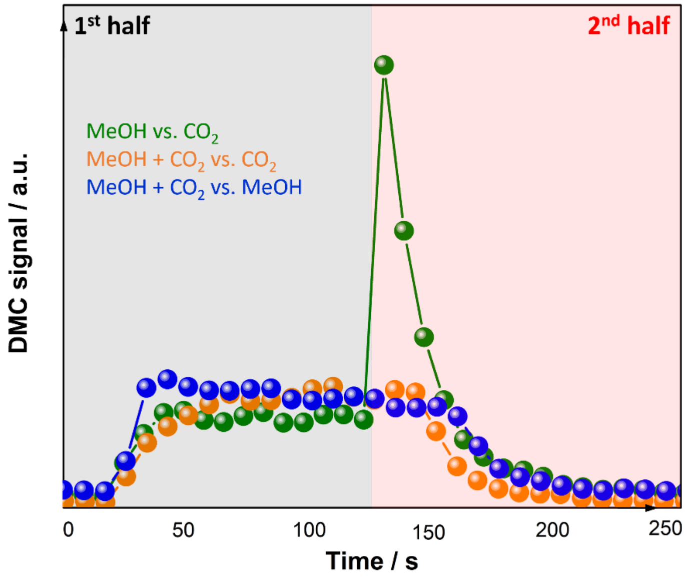

Figure S3 DMC signal measured by MS (m/z = 59) in the three periodic switching experiments of the gas atmosphere, namely (i) MeOH vs. CO_2 (green), (ii) MeOH+CO_2 vs. CO_2 (orange) and (iii) MeOH+CO_2 vs. MeOH (blue), performed at 120 °C over CeO_2.

This indicated methoxy adsorption as the critical step for activation was closely looked into for the cases (i) and (ii) (**Figure S4**). There is a great difference between the concentrations (absorbance) of the methoxy species in the two experiments. Due to the internal background we take, these methoxy species are those reversibly/reactively adsorbed on the surface. Obviously, for case (i) (**Figure S4 A**) when the MeOH and CO_2 flows are fully switched, the concentration of the methoxy species changes more significantly, ca. 4 times of the case (ii) (**Figure S4 B**) in accordance with the gas-phase DMC concentration (**Figure S3**). These results confirm the competitive nature of surface adsorption by CO_2 and MeOH and important surface activation by MeOH adsorption and formation of Ce^{3+}.

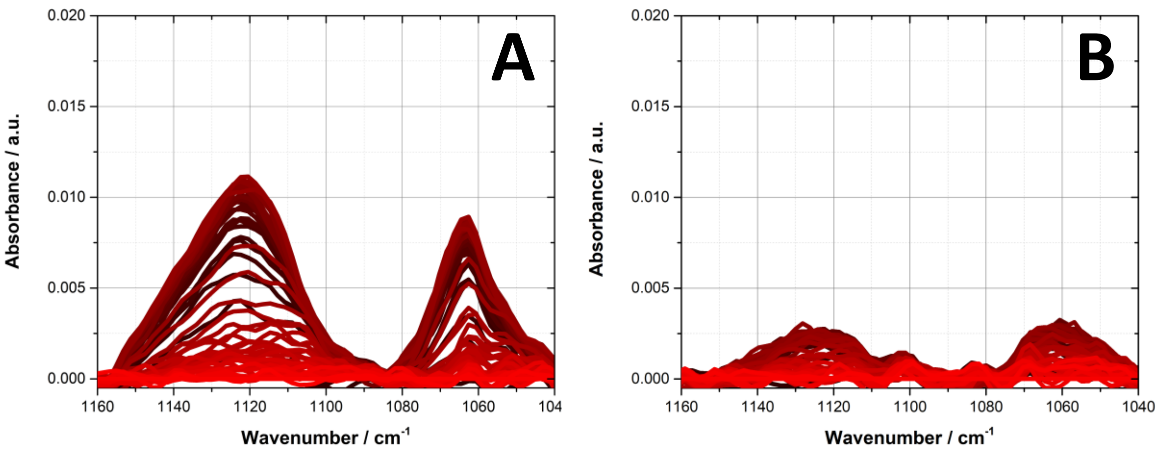

Figure S4 Methoxy bands during the periodic switching experiments in two periodic switching experiments of the gas atmosphere, namely (A) MeOH vs. CO_2, (B) MeOH+CO_2 vs. CO_2, performed at 120 °C over CeO_2.

MES results on the low frequency region from 950 to 1800 cm^{-1}:

The analysis of the time-domain spectra shown in Figure 1 by MES, and the resulting phase-domain spectra are presented in **Figure S5** in comparison to the time-domain spectra (identical to **Figure 1**, but plotted in 1D) for clearer comparison. Although the phase-domain spectra show clearly higher S/N, MES generally suffers from disentangling overlapping bands and the phase-resolved spectra often do not show chemically-pure and meaningful spectra as evident from **Figure S5**. A deeper look into the bridged (ca. 1060 cm^{-1}) and terminal (ca. 1120 cm^{-1}) methoxy bands in the time-domain spectra in **Figure S5** and **Figure 1** shows that there are some changes in the positions and broadness of the bands. The phase-domain spectra (**Figure S5**) show that different species cannot be well-resolved for the two bands. On the other hand, such features are well-resolved in **Figure 2** by multivariate spectral analysis as chemically different species

as described in the manuscript, showing the disentangling power of the multivariate spectral analysis.

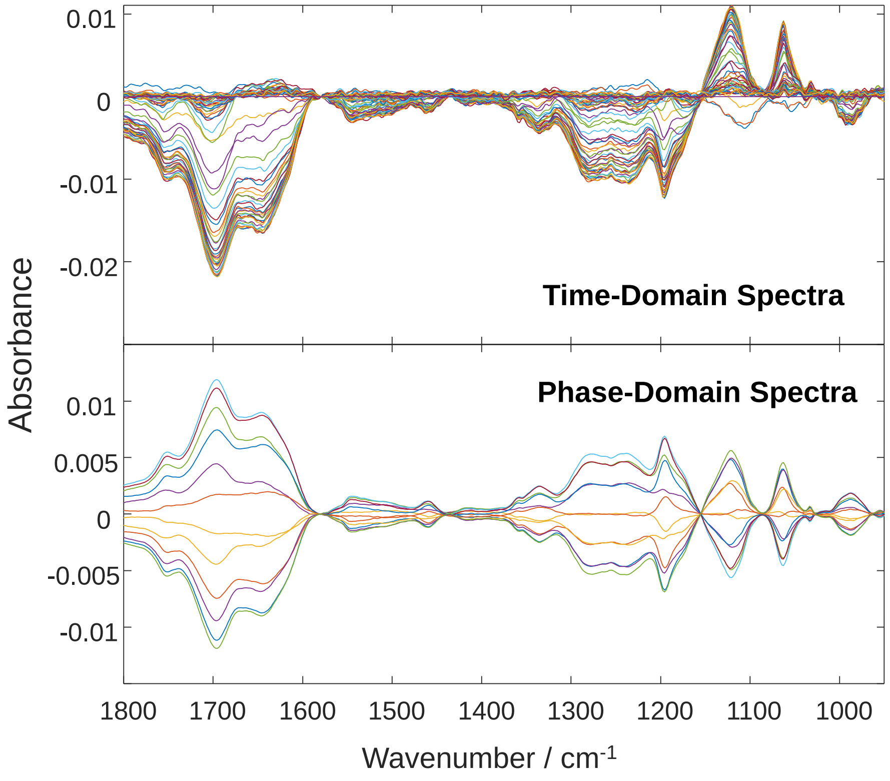

Figure S5 Time-domain and phase-domain spectral analysis of the spectral data presented in **Figure 1**. The phase angles were at 0-350° at the increment of 30°.

DRIFTS spectra of the high frequency region during the MeOH vs. CO_2 concentration perturbation experiment and the corresponding MCR-ALS results:

The DRIFT spectra in the higher frequency region (above ca. 2000 cm^{-1}) under the identical conditions to the experiment shown in **Figure 1** are included as **Figure S6** (for the sake of clarity gas phase CO_2 signal is subtracted). There are changes above 3000 cm^{-1} as broad peak features as expected from the changes in the OH stretching bands of

the surface species. However, this part is not included in the figure since this region seems affected strongly by the reflectivity change due to the surface coverage of methanol and/or water, inducing major baseline changes. Hence, the time-resolved spectra up to ca. 3000 cm^{-1} are shown. The bands at 2800-2900 cm^{-1} are assigned to C-H stretching vibrations of the surface intermediates, although the assignments for the broad band with a peak ca. 2700 cm^{-1} and another small one at ca. 2500 cm^{-1} could not, indubitably, be made.

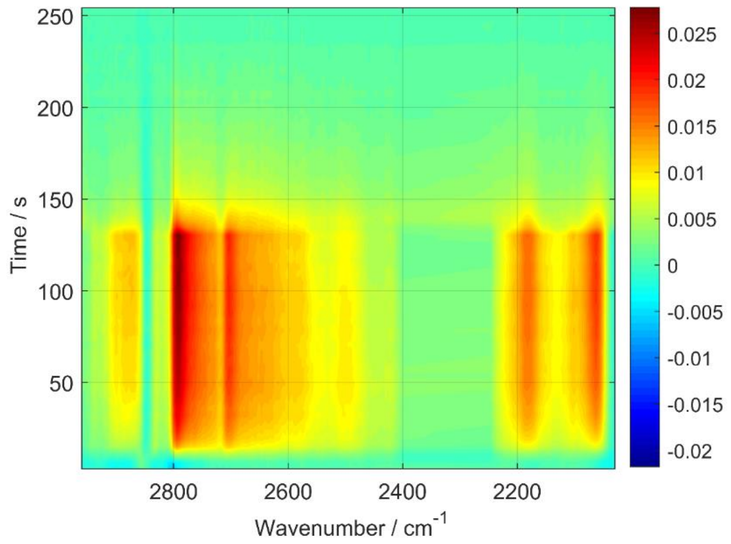

Figure S6 Time-resolved DRIFT spectra in the higher frequency region taken during methanol (the first half period) vs. CO_2 (the second half period) concentration perturbation experiment performed at 120 °C at 7 ml min^{-1}. The DRIFT spectra were calculated taking the last spectrum in the CO_2 atmosphere as background. The gaseous CO_2 signal has been subtracted for the sake of clarity.

Since peculiar changes (possibly delayed and enhanced bands) in the C-H stretching region of **Figure S6** upon switching from MeOH to CO_2 were detected, multivariate spectral analysis for the region of 2400-2950 cm^{-1} was performed and the results are shown in **Figure S7**. The analysis clarified three kinetically distinguishable components.

One of them, Component 2, contains mostly the baseline change, likely due to the reflectivity change, and thus is not discussed further. Interestingly, there are two components (Components 1 and 3) which show similar spectral features but with very different temporal profiles (**Figure S7, right**). Compared to **Figure 2** and the reactivity data, we can safely conclude that Component 1 is assigned to surface methoxy species and Component 3 to mainly monomethyl carbonate (MMC) species. The similar spectral features of Components 1 and 3 are reasonable considering the similarity of the chemical structures of these species.

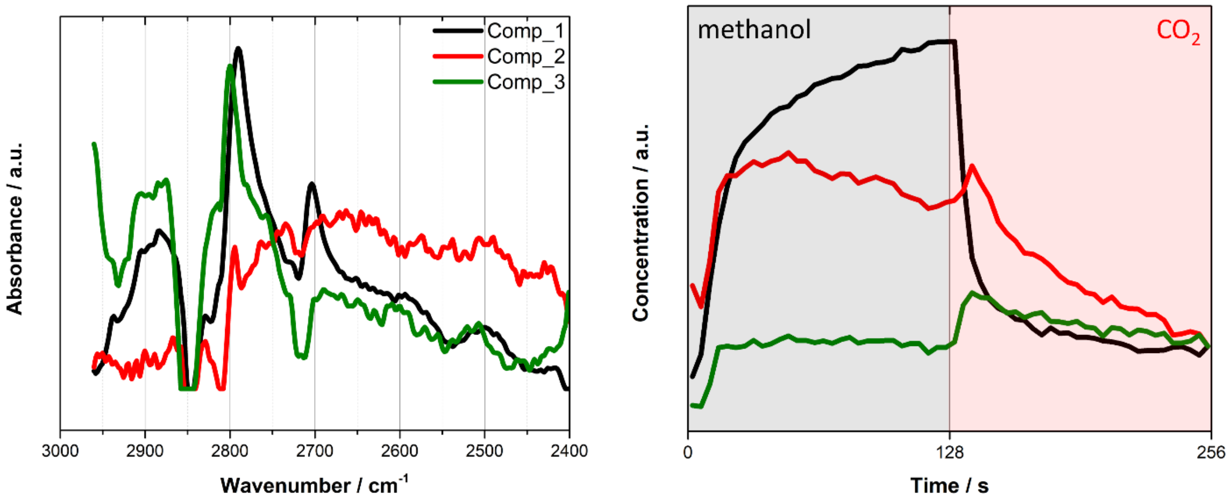

Figure S7 (*left*) Three components spectra and (*right*) the corresponding concentration profiles obtained by multivariate spectral analysis applied on the DRIFT spectra shown in Figure S6 for the region of 2400-2950 cm^{-1}.

Furthermore, interestingly there are two strong bands in the region of 2000-2250 cm^{-1} (**Figure S6**). The interpretation of the bands is not straightforward since no bands are expected in this region from the surface species discussed in the manuscripts or other possible ones. According to Lustemberg *et al.* these bands can be assigned to the reduced Ce sites (Ce^{3+}) created by methanol adsorption (thus methoxy). This reference[15]

indicates that methanol adsorption can induce Ce^{3+} formation in agreement with our Raman study and now this can be confirmed by the band emergence in this vibrational frequency region. This insight of Ce^{3+} formation seems important and affects the formation of DMC as debated throughout the manuscript.

In situ XAS/Raman/MS results

The experiments were performed at the Swiss Norwegian Beam Line (SNBL) at the European Synchrotron Radiation Facility (ESRF) in Grenoble, France. We targeted to follow the changes in the oxidation state of Ce by XAS at Ce K and L3-edges and associated structural change by Raman spectroscopy under the transient conditions (methanol vapor vs. CO_2 flow). **Figures S8** and **S9** present the XANES (X-ray absorption near edge structure) results of the oxidation-reduction cycles performed at 350 °C. **Figure S5** shows the Raman results associated with the same experiment. MCR analysis has been performed to distinguish between Ce^{4+} (under O_2, red) and Ce^{3+} (under H_2, black) states, respectively.

XANES (**Figures S8** and **S9**)*: component 1 (black, Ce^{3+}) and component 2 (red, Ce^{4+})*

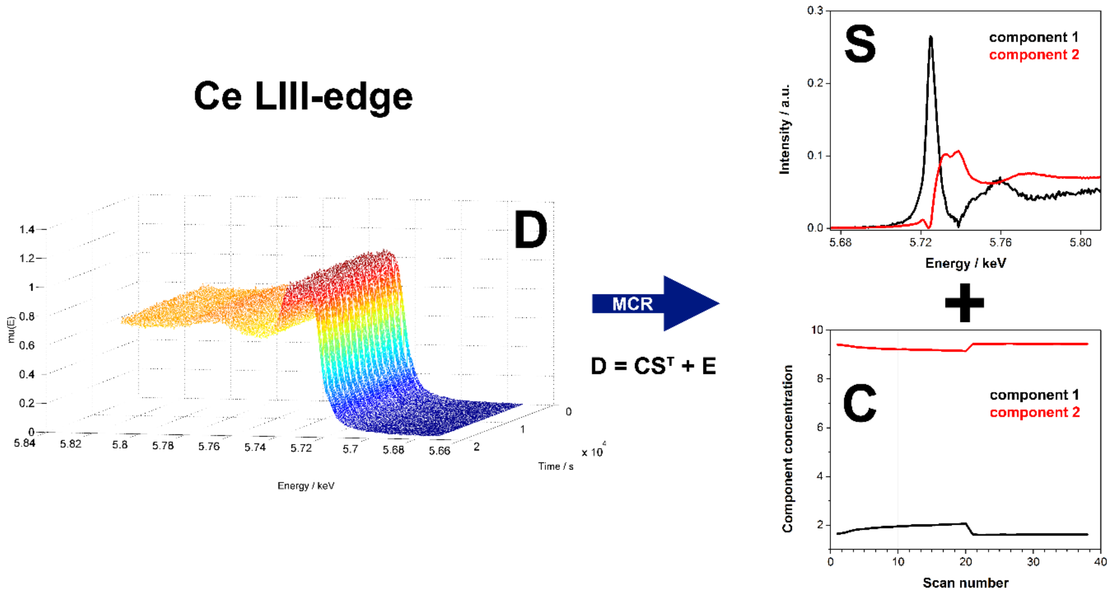

Figure S8 Ce L3-edge: H_2 vs. O_2 concentration perturbation experiment performed at 350 °C. MCR analysis has been applied to disentangle the two component spectra (upper panel, **S**) and the corresponding concentration profiles (lower panel, **C**), respectively.

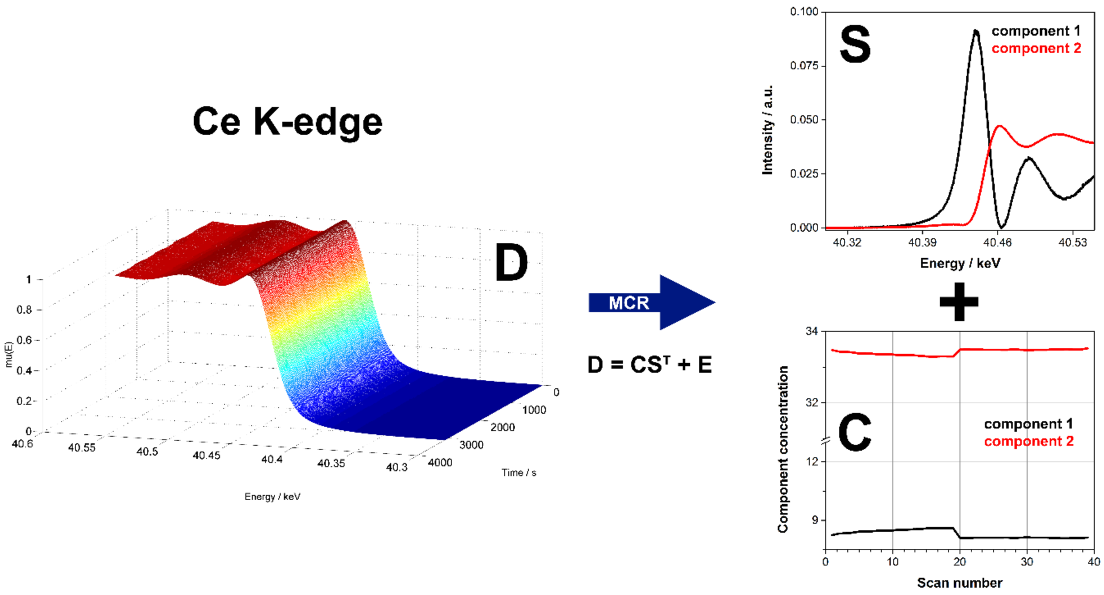

Figure S9 Ce K-edge: H_2 vs. O_2 concentration perturbation experiment performed at 350 °C. MCR analysis has been applied to disentangle the two component spectra (upper panel, **S**) and the corresponding concentration profiles (lower panel, **C**), respectively.

*Raman (**Figures S10**): component 1 (red, Ce^{4+}) and component 2 (black, Ce^{3+})*

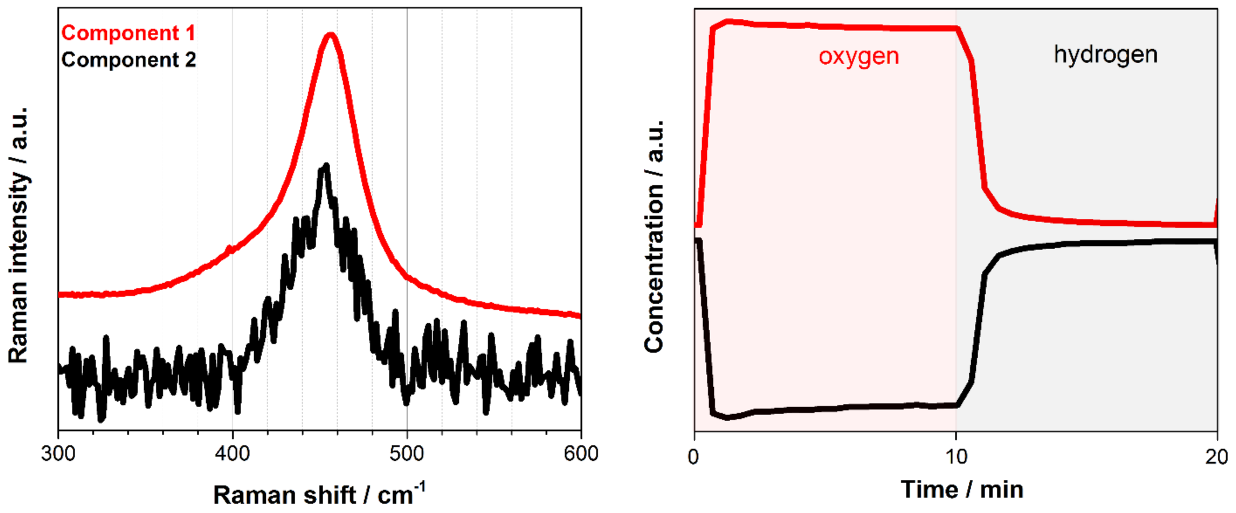

Figure S10 MCR applied on the region of F_{2g} band of CeO_2. Raman spectra were collected for a H_2 vs. O_2 concentration perturbation experiment performed at 350 °C. Raman measurements were done with 532 nm laser. Both spectra (*left*) and concentration (*right*) profiles are normalized for clarity.

DMC synthesis. CO_2 vs. methanol at 120 °C, 785 nm Raman laser (red)

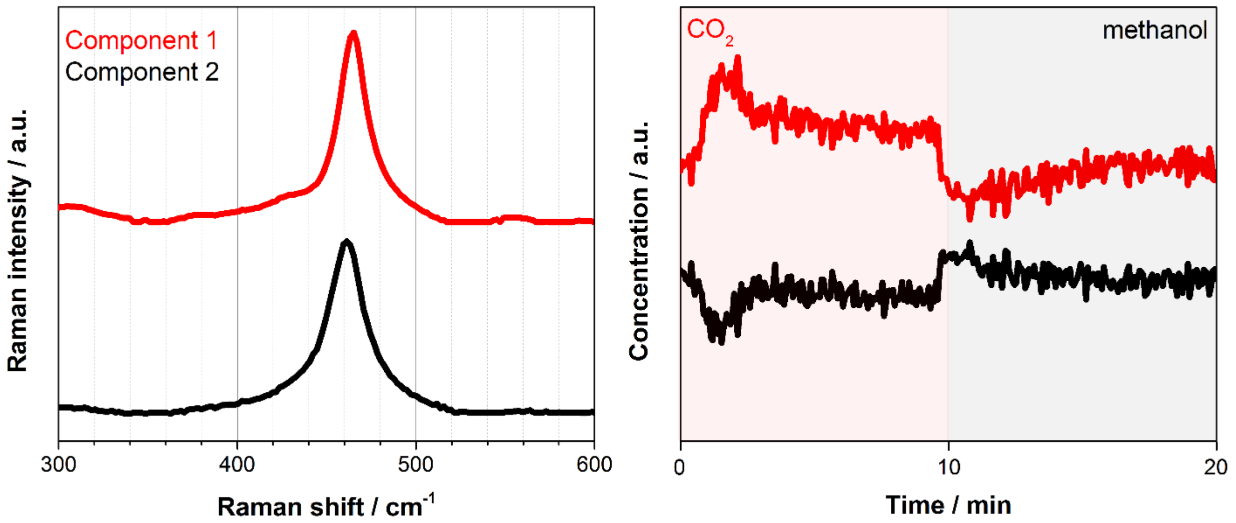

Figure S11 MCR applied on the region of F_{2g} band of CeO_2. Raman spectra were collected during CO_2 vs. methanol concentration perturbation experiment performed at 120 °C. Raman measurements were performed with 785 nm laser.

DFT results and discussion

Adsorption Structure

The adsorption structures of CO_2, methanol, and monomethyl carbonate species over CeO_2(111) are examined in this section.

A. CO_2

Three stable adsorption structures for CO_2 are identified by geometry optimization, and these structures are shown in **Figure S12 (a-c)**. Two of them are recognized by surface carbonate species involving a covalent bond between the carbon and surface oxygen atoms, and they are referred to as monodentate and bidentate structures, respectively. Adsorption energies of these structures are calculated to be −52 and −29 kJ/mol, respectively, and these values are in good agreement with the previous works by Wang and coworkers (0.52 eV for monodentate structure).[16] The other stable structure is a molecularly adsorbed structure with an adsorption energy of −15 kJ/mol, which is much smaller than those of surface carbonate species.

B. Methanol

Adsorption structures of a methanol molecule over the CeO_2 surface has been investigated in our previous work, and for details see reference.[17] In short, two optimized structures, molecular and dissociative states, were obtained (**Figure S12 (d-e)**), and the adsorption energies are −57 kJ/mol and −66 kJ/mol for the molecular and dissociative state, respectively, and the dissociative adsorption is favorable on the surface due to the high basicity of surface oxygen atoms.

C. Monomethyl carbonate (MMC)

Several stable adsorption structures are identified for monomethyl carbonate species over CeO_2. **Figure S12 (i)** shows the most stable adsorption structure (**DB**) where MMC is dissociatively-adsorbed with an adsorption energy of 113 kJ/mol and two oxygen atoms interact with the surface Ce atoms. The adsorption structures shown in **Figure S12 (f-h)** (**DM1**, **DM2**, and **DM3**) also exhibit dissociative state, and in these structures, only one oxygen atom is directly interacting with the surface Ce atom. The adsorption energy of **DM1** is very similar to that of **DB**. The third type of the adsorption structure (**MMC_S**) is the surface carbonate species where the carbon atom and surface oxygen atoms form a covalent bond, and this structure is the least stable structure with an adsorption energy of −54 kJ/mol.

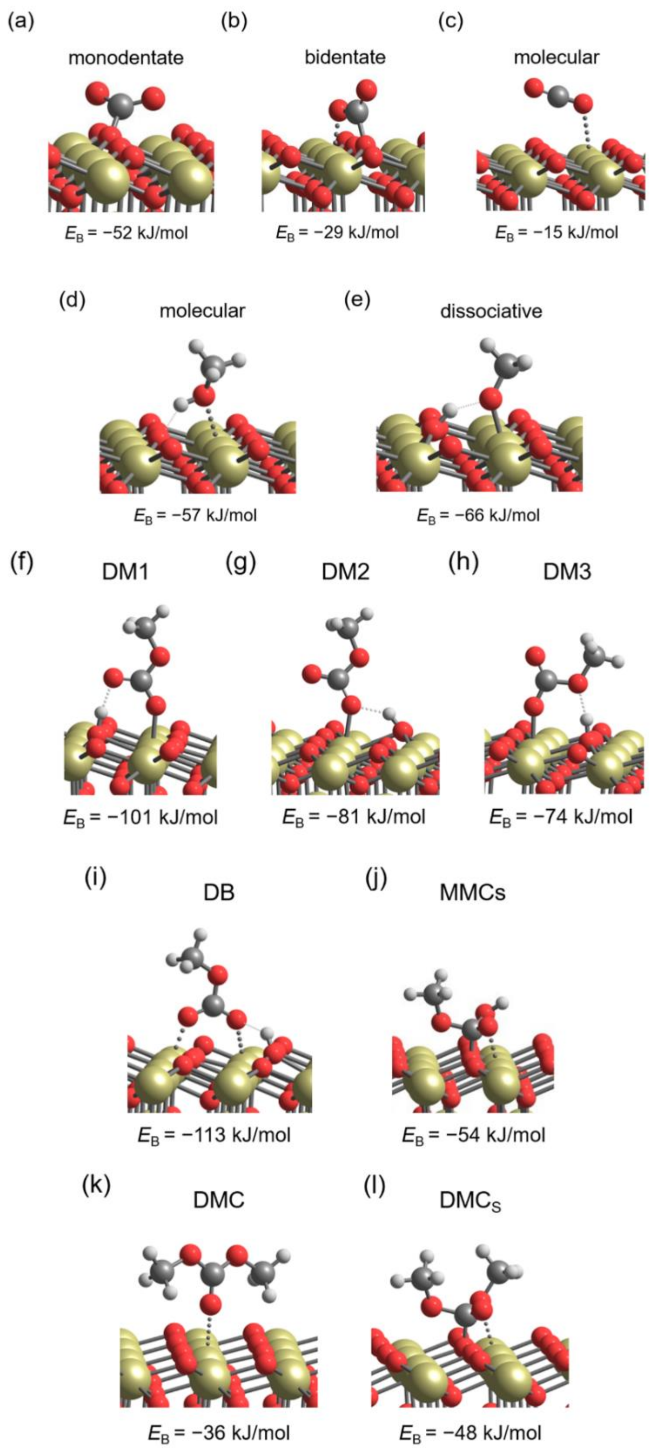

Figure S12 Adsorption structures (a-c) CO_2, (d-e) methanol, (f-j) monomethyl carbonate, (i-j) DB and MMCs, and (k-l) dimethyl carbonate (DMC) on the CeO_2(111) surface. The atoms are color-coded as follows: gray, H; dark gray, C; red, O; dark yellow, Ce.

Computational details

DFT calculations. All DFT calculations were carried out by the spin-polarized periodic-boundary DFT with the mixed Gaussian and plane-waves (GPW) approach implemented in the CP2K program package.[18] The double-ζ valence plus polarization (DZVP) basis sets of the MOLOPT type[19] were employed for H, C, O atoms to represent the valence electrons, and the norm-conserving Goedecker-Teter-Hutter pseudopotentials[20,21] were used to describe the interactions between the valence and core electrons. For Ce atoms, we employed the basis sets and pseudopotential generated by Wang and coworkers.[16] The energy cutoff of 400 Ry was taken for the auxiliary plane wave expansion of the density. The generalized-gradient approximation by the Perdew−Burke−Ernzerhof (PBE) functional[22] models was employed as the exchange and correlation potential, and the DFT+U approach[23,24] was used in order to correctly represent the nature of 4*f* orbitals of Ce atoms, where the occupancies of 4*f* orbitals were calculated by Mulliken population analysis. The U value is set to 7.0 eV following the previous works.[25] The Brillouin zone integration was performed with a reciprocal space mesh consisting of only the Γ-point in the GPW approach. For geometry optimization, the forces on all atoms are minimized to less than 0.02 eV/Å (4.5×10^{-4} hartree/bohr).

The surface of $CeO_2(111)$ is modeled as a periodic $p(3\times3)$ hexagonal slab of 27 CeO_2 unit with three O-Ce-O tri-layers. The dimensions of a simulation cell are set to $a = b =$ 11.56, c = 25.0 Å and $\alpha = \beta = 90$, $\gamma = 60$ degrees, and this slab is separated by ~16 Å of vacuum space in the direction perpendicular to the surface. The lattice parameters of the $CeO_2(111)$ slab are determined by the cell optimization of the bulk CeO_2, and the optimized lattice constant of 5.450 Å is in good agreement with the experimental value of

5.411 Å.[26,27] The bottom O-Ce-O tri-layers are fixed at the bulk positions during the geometry optimization and also in molecular dynamics simulations. In calculating the energies of gas-phase molecules, the simulation cell of $a = b = c = 15.0$ Å in the cubic box is used.

The adsorption energy of a molecule E_A to the CeO_2(111) surface is calculated according to:

$$E_{\mathrm{A}} = E_{\mathrm{mol+surf}} - E_{\mathrm{mol}} - E_{\mathrm{surf}} \qquad (0.1)$$

where $E_{\mathrm{mol+surf}}$ is the total electronic energy of surface-molecular system while E_{surf} and E_{mol} are the energies of pristine surface and an isolated molecule, respectively. In this definition, the more negative value of adsorption energy indicates a stronger binding to the surface.

Molecular dynamics simulations. In the molecular dynamics simulations, the simulation cell size is set to be the same as above, and for modeling the complex reactions involving several methanol molecules, one CO_2 and eight methanol molecules are placed over the CeO_2 surface to represent the adsorbed methanol monolayer. The simulations do not take into account the effect of water molecules that may dissociatively adsorb on the surface because the reaction mechanism and activation energies are not affected by the presence of dehydrating agent (2-cyanoypridine) that reacts smoothly with the water molecules.[29] Molecular dynamics simulations are performed with the canonical ensemble (NVT) condition, where the temperature is controlled at 360 K by the Nóse-Hoover thermostats. The mass of hydrogen is replaced with that of deuterium allowing for a larger time step of 1.0 fs. The free energy profiles are estimated by the blue moon

ensemble method,[28,30] and the reaction coordinates used to obtain free energy profiles are given in **Table S1**, where a distance between two atoms is used. The grid widths for performing constrained molecular dynamics simulations ranges from 0.1 to 0.5 Å, depending on the distance between the two atoms. The free energy of the reaction from the initial state r_a to the final state r_b is given by:

$$\Delta F = \int_{r_a}^{r_b} \langle \lambda_r \rangle dr \qquad (0.2)$$

where λ_r is a Lagrange multiplier associated with the parameter ξ used in the SHAKE algorithm.[31] After equilibration of the system is reached, a total of 20 ps simulations are performed for each reaction coordinate to obtain the above averages.

Table S1 Reaction coordinate used in the blue moon ensemble method.

	Reaction step	ξ
Path A	**CO_2* → CO_2 (m)**	$r(O_S–C)$
	**CO_2 (m) → MMC_S**	$r(C–O_M)$
Path B	**CH_3O^-* → MMC**	$r(C–O_M)$
	**MMC →MMC_S**	$r(C–O_S)$
	**MMC_S → INT_1**	$r(C–O_{OH})$
	**INT_1 → INT_2**	$r(C–O_M)$
	**INT_2 → DMC***	$r(O_S–C)$

O_S: surface oxygen atom, O_M: oxygen atom in the methoxy species, O_{OH}: oxygen atom of the hydroxyl group.

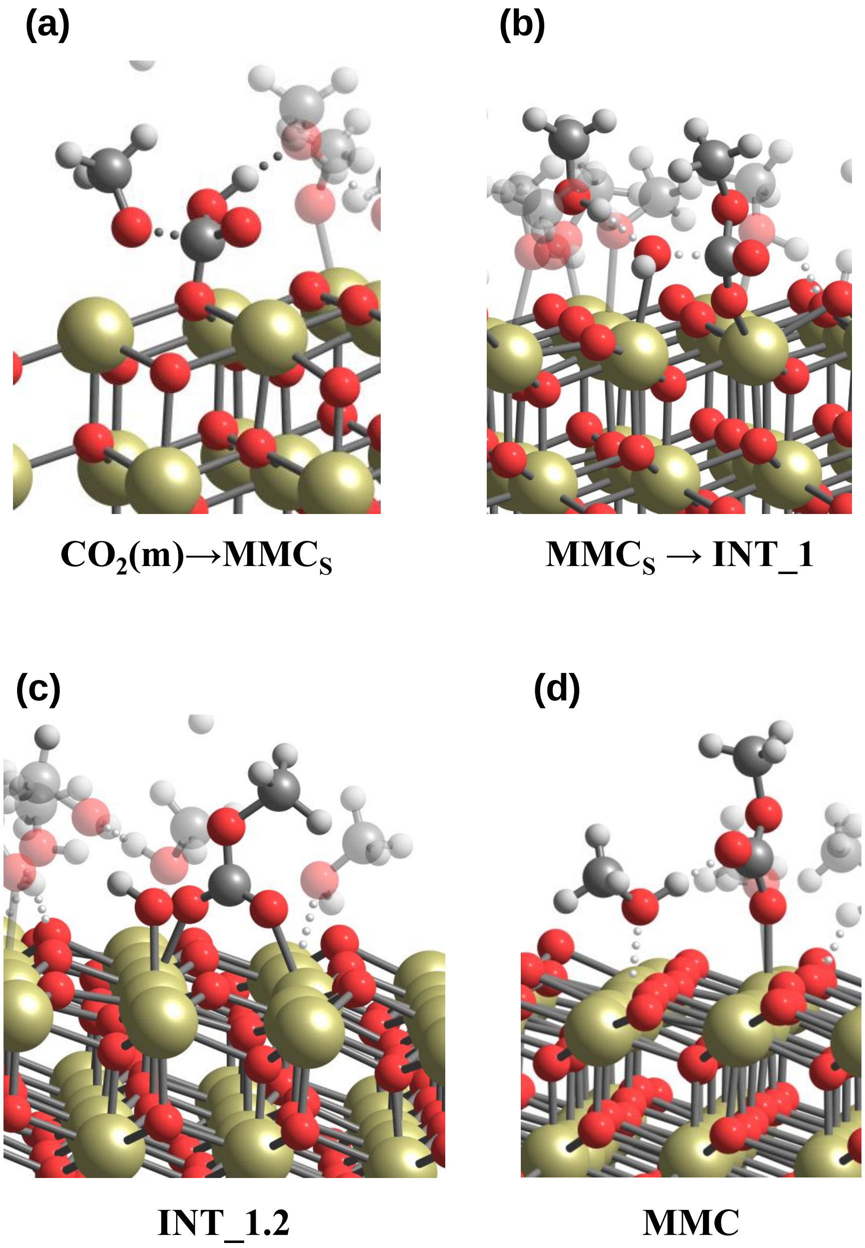

Figure S13 Snapshots of the MD trajectory near the transition state structure of (a) **CO_2(m)→MMC_S** and (b) **MMC_S → INT_1**, and configuration at (c) **INT_1.2** and (d) **MMC**.

References

1 A. Voronov, A. Urakawa, W. van Beek, N. E. Tsakoumis, H. Emerich and M. Rønning, *Analytica Chimica Acta*, 2014, **840**, 20–27.
2 SNBL@ESRF, https://www.snbl.eu/.
3 W. van Beek, O. V. Safonova, G. Wiker and H. Emerich, *Phase Transitions*, 2011, **84**, 726–732.
4 A. Urakawa, N. Maeda and A. Baiker, *Angewandte Chemie International Edition*, 2008, **47**, 9256–9259.
5 A. Malik, A. de Juan and R. Tauler, in *40 Years of Chemometrics – From Bruce Kowalski to the Future*, American Chemical Society, 2015, vol. 1199, pp. 95–128.
6 R. Tauler, A. Smilde and B. Kowalski, *Journal of Chemometrics*, 1995, **9**, 31–58.
7 R. Tauler, *Chemometrics and Intelligent Laboratory Systems*, 1995, **30**, 133–146.
8 R. Tauler, *Journal of Chemometrics*, 2001, **15**, 627–646.
9 M. Amrhein, B. Srinivasan, D. Bonvin and M. M. Schumacher, *Chemometrics and Intelligent Laboratory Systems*, 1996, **33**, 17–33.
10 J. Saurina, S. Hernández-Cassou, R. Tauler and A. Izquierdo-Ridorsa, *Journal of Chemometrics*, 1998, **12**, 183–203.
11 A. de Juan and R. Tauler, *Analytica Chimica Acta*, 2003, **500**, 195–210.
12 J. Jaumot, R. Gargallo, A. de Juan and R. Tauler, *Chemometrics and Intelligent Laboratory Systems*, 2005, **76**, 101–110.
13 A. de Juan, J. Jaumot and R. Tauler, *Anal. Methods*, 2014, **6**, 4964–4976.
14 J. Jaumot, A. de Juan and R. Tauler, *Chemometrics and Intelligent Laboratory Systems*, 2015, **140**, 1–12.
15 P. G. Lustemberg, M. V. Bosco, A. Bonivardi, H. F. Busnengo and M. V. Ganduglia-Pirovano, *J. Phys. Chem. C*, 2015, **119**, 21452–21464.
16 Y.-G. Wang, D. Mei, J. Li and R. Rousseau, *J. Phys. Chem. C*, 2013, **117**, 23082–23089.
17 M. Tamura, R. Kishi, A. Nakayama, Y. Nakagawa, J. Hasegawa and K. Tomishige, *J. Am. Chem. Soc.*, 2017, **139**, 11857–11867.
18 J. VandeVondele, M. Krack, F. Mohamed, M. Parrinello, T. Chassaing and J. Hutter, *Computer Physics Communications*, 2005, **167**, 103–128.
19 J. VandeVondele and J. Hutter, *J. Chem. Phys*, 2007, **127**, 114105.
20 S. Goedecker, M. Teter and J. Hutter, *Phys. Rev. B*, 1996, **54**, 1703–1710.
21 C. Hartwigsen, S. Goedecker and J. Hutter, *Phys. Rev. B*, 1998, **58**, 3641–3662.
22 J. P. Perdew, K. Burke and M. Ernzerhof, *Phys. Rev. Lett.*, 1996, **77**, 3865–3868.
23 J. F. Herbst, R. E. Watson and J. W. Wilkins, *Phys. Rev. B*, 1978, **17**, 3089–3098.
24 S. L. Dudarev, G. A. Botton, S. Y. Savrasov, C. J. Humphreys and A. P. Sutton, *Phys. Rev. B*, 1998, **57**, 1505–1509.
25 M. Farnesi Camellone, F. Negreiros Ribeiro, L. Szabová, Y. Tateyama and S. Fabris, *J. Am. Chem. Soc.*, 2016, **138**, 11560–11567.
26 L. Gerward and J. S. Olsen, *Powder Diffraction*, 1993, **8**, 127–129.
27 A. Nakajima, A. Yoshihara and M. Ishigame, *Phys. Rev. B*, 1994, **50**, 13297–13307.
28 M. Sprik and G. Ciccotti, *The Journal of Chemical Physics*, 1998, **109**, 7737–7744.
29. Honda, M. Tamura, Y. Nakagawa, K. Nakao, K. Suzuki and K. Tomishige, *J. Catal.*, 2014, **318**, 95–107.
30. E. A. Carter, G. Ciccotti, J. T. Hynes and R. Kapral, *Chem. Phys. Lett.*, 1989, **156**, 472–477.
31. J.-P. Ryckaert, G. Ciccotti and H. J. C. Berendsen, *J. Comput. Phys.*, 1977, **23**, 327–341.
